# Application of Quantitative Microbiology and Challenge Tests to Reach a Suggested Food Safety Objective in a Middle Eastern-Style Ready-to-Cook Chicken Product

**DOI:** 10.3390/foods11131900

**Published:** 2022-06-27

**Authors:** Tareq M. Osaili, Vasiliki Giatrakou, Athina Ntzimani, Maria Tsiraki, Ioannis N. Savvaidis

**Affiliations:** 1 Department of Clinical Nutrition and Dietetics, College of Health Sciences, University of Sharjah, Sharjah P.O. Box 27272, United Arab Emirates; tosaili@sharjah.ac.ae or; 2 Department of Nutrition and Food Technology, Jordan University of Science and Technology, Irbid 22110, Jordan; 3 Hellenic Research and Innovation Center, Leoforos Kifisou 128, 121 31 Athens, Greece; vgiatrakou@hriclabs.gr or; 4 Laboratory of Food Chemistry and Food Microbiology, Department of Chemistry, University of Ioannina, 451 10 Ioannina, Greece; ntzimani@chemeng.ntua.gr (A.N.); tsir_maria@yahoo.gr (M.T.); 5 Laboratory of Food Chemistry and Technology, School of Chemical Engineering, National Technical University of Athens, 157 72 Athens, Greece; 6 Dairy Research Department, Institute of Technology of Agricultural Products, General Directorate of Agricultural Research, ELGO-DIMITRA, Katsikas, 452 21 Ioannina, Greece; 7 Department of Environmental Health Sciences, College of Health Sciences, University of Sharjah, Sharjah P.O. Box 27272, United Arab Emirates

**Keywords:** middle eastern food, risk evaluation, natural antimicrobials, chitosan, thyme oil, predictive models

## Abstract

The contamination of ready-to-eat (RTE) and ready-to-cook (RTC) food products is a major global issue raising worry to consumers. Therefore, the behavior of *Listeria monocytogenes* and *Salmonella* spp., inoculated on a traditional Middle Eastern (M.E.) ready-to-cook (RTC) chicken product (“Taouk”-style), using the Risk Ranger^®^ tool and the necessary management options (to accomplish the hypothetical food safety objectives (FSO)), when unsuspecting consumers may taste such a product were the primary subjects of our study. The behavior of the aforementioned pathogens was studied in the presence and absence of a selected natural antimicrobial combination (chitosan [CH] and thyme oil [T]), and were added as a combined treatment (M-CH-T) to the RTs chicken samples, stored at 4 or 8 °C for a period of 8 d. In the product, wherein no antimicrobials were added (control treatment, M), the initial counts of *L. monocytogenes* increased by ca. 1.5 (4 °C) and 3.0 (8 °C) log colony-forming units (CFU)/g during an 8-d storage. *Salmonella* spp. numbers did not increase during storage at 4 °C in the non-treated product, but at 8 °C, an increase of ca. 2.5 log CFU/g occurred. Addition of CH in combination with T to the RTC product (M-CH-T) inhibited the growth of *L. monocytogenes* and produced lower counts of *Salmonella* at 4 °C. However, M-CH-T treatment was less effective against both pathogens compared to the control after the 6th day of storage (8 °C). Predictive models based on quantitative microbiology, combined with hazard identification applied in the present study, may be potential means of assessing the safety of the RTC chicken products. It must be noted that for warranting the food safety of especially perishable items (e.g., chicken products), an efficient food safety management system must be applied, in addition to testing of the finished product, (e.g., based on the HACCP principles).

## 1. Introduction

During the last few years, quicker meals such as Ready-to-Eat or Cook (RTE or RTC) food products have gained ground, owing to the shorter preparation time needed, ease of use, and freshness [1]. Food contamination with pathogenic bacteria may pose a serious health risk to the consumer. The presence and prevalence of foodborne pathogens in food products is a great concern for human health, with many outbreaks of illnesses caused annually [2,3,4,5]. RTC products are usually meals that do not include a pasteurization step during their production, therefore they must be cooked by heating before they are consumed. This cooking step, which is performed by the consumer, is considered crucial for the microbiological safety of the products. In our study, a traditional M.E. poultry, kebab-type chicken product (“Taouk”-style) was selected as the food model. Vazgecer et al. [6] reported that raw kebab meals are products that require adequate cooking to ensure their safety. The demand for minimally processed foods made of natural ingredients is steadily increasing [7,8]. Essential oils (EOs, e.g., oregano or thyme), which are “Generally Recognized as Safe” (GRAS) food additives, can be applied either with or without other preservative “hurdle” technologies (e.g., packaging/intelligent packaging, high pressure, irradiation, etc.) with the view to decreasing microbial counts, reducing microbial growth rates and increasing lag phases of the spoilage microorganisms, preventing the potential growth of pathogens in perishable foods (poultry, dairy, fish/seafood, etc.) [9,10,11].

“Taouk”-style poultry products, which may consist of either fresh chicken chunks and/or with chopped bell peppers, are usually marinated and are popular in the Levant countries [12] and also in the Eastern Mediterranean region (Lebanon, Greece, Cyprus and Turkey). These RTC products, which are usually stored refrigerated (4 °C), may support the growth of psychrotrophic bacteria, such as pseudomonads or *Brochothrix thermosphacta*, consequently leading to spoilage of these products. Traditionally, the processing (cutting of chicken meat to pieces, raw bell peppers, etc.) and the final preparation of such RTC chicken products is manually conducted on skewers; therefore, if no strict hygiene measures are taken, such practices may increase the potential for cross-contamination. Additionally, many factors during processing or consumption, such as temperature abuse during processing or retail prolonged storage, poor manufacturing practices, and potentially high (initial) levels of microbiota in the raw material, could lead to the presence of both undesirable bacteria that cause spoilage and the occurrence of foodborne bacteria, which are pathogenic, e.g., *Listeria monocytogenes* and *Salmonella* spp., in such products. According to Mor-Mur and Yuste [13], pathogens that are most likely to be found in poultry products (chicken and turkey) include *Salmonella* spp., *Campylobacter*, *Arcobacter*, *Listeria monocytogenes*, *Yersinia enterocolitica*, and *Aeromonas hydrophila*. If there is an abuse of temperature, outgrowth and toxin formation can be a threat. *Clostridium botulinum*, *Clostridium perfringens*, *Bacillus cereus*, and *Staphylococcus aureus* are the most dangerous toxin-producing bacteria for these types of products. In fresh vegetables, the prevailing microorganisms (usually on the surface of produce) are mainly Gram-negative saprophytes, but there is a possibility of pathogenic bacteria (e.g., *Yersinia enterocolitica*, *Salmonella*, enteropathogenic *Escherichia coli*, implicated in foodborne incidents) [14,15], as well as Gram-positive bacteria (*Bacillus cereus*, *Clostridium* spp., and *L. monocytogenes*, which are found in soil) being present [15]. Slicing vegetables is a procedure that might result in microbiological deterioration of the product due to the release of nutrients, which leads to the growth of post-processing microorganisms [15].

During the preparation of the “Taouk”-style RTC chicken product, a processing step that inactivates pathogens is not included; therefore, it is imperative to determine the level of the risk that these pathogens pose at the point of consumer consumption.

Within the past 20 years, Codex Alimentarius has set up a risk analysis framework and recent progress in this area (Food Safety and Risk Assessment) has resulted in a valuable tool, the application of which is now legally required [16,17,18]. A strong need has arisen to implement government policies; efforts and measures have been made and taken to reduce foodborne illnesses but, up until now, such measures have been difficult to enforce in practice. The International Commission on Microbiological Specifications for Foods [19] in this context initiated and proposed a series of food safety objectives (FSOs). A food safety objective (FSO) denotes the maximum permissible level of a microbiological hazard in a food commodity at the moment of consumption and is based purely on management decisions, regarding the threshold (acceptable) risk of that hazard to the population or on a public health goal [20]. Codex defines FSO as the maximum frequency and/or concentration of a hazard in a food at the time of consumption that reassures or contributes to the appropriate level of protection (ALOP) [16,17].

The ALOP is an expression related to a population’s health, which may also be defined as the goals that any country sets in order to protect the life of humans, animals and plants from hazards, as reflected in legislation and other official documents, policies and procedures aiming to determine the frequency and level of hazards, rather than eliminating the hazards [21]. FSOs have provided a link between public health and performance objectives (PO), microbiological standards/criteria, etc., as well as a more objective means of establishing a stringency of food control systems [21]. A PO is a required outcome of a step or could be applied as a combination of operations ensuring that an FSO is met.

The establishment of the values of both ALOP and FSO is based mainly on human health and the competent authorities’ decisions. However, until now, there has been no available information of the existence of a country that has established ALOP and FSO values to ensure operational food safety management, which may be attributed to the inability of governments to define public health priorities in the form of ALOP values and relate them to FSOs. Establishing an FSO for a specific hazardous agent is based on information obtained from danger characterization (understanding of the relationship of the dose–response for healthy and sensitive populations), knowledge of the hazard, risk evaluation by an expert panel, quantitative microbial risk assessment, and challenge tests [19,20,22].

Many researchers in the existing literature have employed a variety of different ALOP and FSO models reporting interesting data [22,23,24,25,26,27], concluding that such concepts may be understood if real case studies are only conducted, as such data may facilitate the practical interpretation of these models [21].

The objectives of the present work were: (i) to identify and prioritize pathogen risks in a traditional M.E. RTC product (“Taouk”) using the Risk Ranger^®^ tool; (ii) to study the survival/growth of two selected pathogens (*L. monocytogenes* and *Salmonella enterica* subsp. *enterica* serovar *Montevideo*) on the RTC product, packaged under a modified atmosphere (MAP) with or without added natural antimicrobials (in our study, we used chitosan (CH)/thyme oil (T) in combination at chilled (4 °C) and abuse temperature (8 °C) conditions); (iii) to test the performance of two predictive models available (Combase^®^ and Gamma model); and (iv) to formulate the necessary management options for the control of these two pathogens and achieve the hypothetical FSO in the consumption scenario.

## 2. Materials and Methods

### 2.1. The Product

The product in the present study is a freshly produced (manually) RTC chicken product (with cut bell pepper chunks), supplied by a local poultry processing company (PINDOS, S.A., Ioannina, Greece). The RTC products (ca. 125 ± 10 g) were transported to the laboratory in insulated polystyrene boxes with ice within 1 h after preparation at the of poultry processing plant. The RTC product consisted of fresh chicken pieces and chopped (chunks) bell peppers, prepared manually and finally fixed on a wooden stick (skewer). The chicken skewers were subsequently packaged under a modified atmosphere, with a gas composition of 30% CO_2_/70% N_2_ (PBI-Dansensor, Ringsted, Denmark), simulating the commercial packaging conditions of the product in the retail supermarkets. The product was heat-sealed using a BOSS N48 packaging machine (BOSS, Bad Homburg, Germany) connected to a gas mixer. A low-density polyethylene/polyamide/low density polyethylene (LDPE/PA/LDPE) packaging material was used. The pouches (VER PACK, Thessaloniki, Greece) were 75 μm in thickness with an O_2_ permeability of 52.2 cm^3^/m^2^/day/atm (relative humidity; 75%, 23 °C) a CO_2_ permeability of 191 cm^3^/m^2^/day/atm (relative humidity 0%, 23 °C) and a water vapor permeability of 2.4 g/m^2^/day (relative humidity; 100%, 23 °C)^.^ After packaging the RTC samples were stored in a temperature-controlled cooling incubator at 2 °C (Sanyo, Osaka, Japan) until the addition of natural antimicrobials and inoculation of pathogens were performed.

### 2.2. Semi-Quantitative Risk Assessment and Quantitative Microbiology

In our study, for the identification and selection of the pathogens presenting the highest risks, likely to be involved in an unsafe RTC product, The Risk Ranger software [28,29] was employed, as also adopted in relevant studies [22,29]. A combination of tools (Combase^®^, Bigelow model and Gamma model) was used to predict the fate/growth or survival of the microorganisms (*L. monocytogenes* and *Salmonella S. enterica* serovar *Montevideo*, selected in our study) in the RTC product, as previously applied [22]. The initial level for growth was 3 log CFU/g, and the inactivation level was 6 log CFU/g. The variables selected were pH of 6.8, a_w_ of 0.99, and temperatures of 4 °C and 8 °C for growth. A temperature abuse (8 °C) was chosen in our study as opposed to 12 °C, given that *Salmonella* spp. are able (and known) to grow even at 7 °C.

### 2.3. Challenge Tests

To validate the predictive models, challenge tests [22] were performed on the RTC product in cases where antimicrobials were either applied or not (see below). The growth of the pathogens selected in the present study was determined during the 6-day product’s sustainability, as well as during a 2-day expansion after the expiration date of the RTC product (8 d).

### 2.4. Inoculum Preparation of Pathogenic Bacteria

*L. monocytogenes* (Scott A, WT) and *S. enterica* subsp. *enterica* serovar *Montevideo* bacterial strains were kindly provided by the Laboratory of Food Microbiology of Wageningen University (the Netherlands). Cultures were subsequently kept at −40 °C on sterile Brain Heart Infusion (BHI) broth (Merck, Darmstadt, Germany) with 20% glycerol added to it. For the activation of cultures, individual transfers of 100 μL of the frozen cultures into 10 mL tubes containing sterile BHI broth (Merck, Germany) were conducted and the resulting mixtures were incubated at 37 °C for 24 h. Working cultures were freshly prepared and stored under refrigeration (4 °C) on agar slants on BHI Agar, sub-cultured monthly. Fully grown cultures (*L. monocytogenes* and *S. Montevideo*) were serially diluted in freshly prepared (sterile) buffered peptone water (0.1% *w*/*v*; pH = 7.0) to obtain a final cell concentration of 10^5^ CFU/mL]. An aliquot of the inoculum (1 mL) was added to the surface of the RTC product to obtain a final cell concentration of approximately 10^3^ CFU/g using a micropipette. Once inoculated, samples were kept at room temperature (ca. 15 min) to allow the inoculum to be absorbed onto the product and, thus, achieve adequate bacterial attachment.

### 2.5. Preparation of Antimicrobials and Packaging-Incubation of the RTC Product

Two antimicrobials were used: CH (Aldrich, Athens, Greece) and T (Mane Fils, Le Bar-sur-Loup, France). Chitosan has a low molecular weight with a moisture content of ≥10% and deacetylation of 75–85%. A stock of 2% wt/vol was obtained by mixing 2 g of CH in 100 mL of 1% (wt/vol) glacial acetic acid and stirring overnight at room temperature. Thyme essential oil (*Thymus vulgaris*) was used in its pure form, consisting of thymol (57.7%), p-cymene (18.7%), and carvacrol (2.8%). Both chitosan and thyme were applied to the inoculated RTC product. An RTC sample (125 ± 10 g) was enclosed aseptically into an open low-density polyethylene/polyamide/low-density polyethylene (LDPE/PA/LDPE) pouch, followed by CH spraying directly onto the product using a micro-spray (final concentration on the product = 1.5% vol/wt), whereas T was added undiluted using a micropipette (final concentration on the product = 0.2% vol/wt). Samples were packaged under a modified atmosphere (as described previously) and were incubated at 4 ± 0.5 °C or 8 ± 0.5 °C, representing chilled and abuse temperatures, for a period of 8 d. In our study, two treatments were tested and included: Control treatment (designated as “M”), which included RTC samples inoculated with the two pathogens, also packaged under a modified atmosphere (as previously) without the addition of antimicrobials, simulating the commercial storage of the RTC product. The test treatment (designated as “M-CH-T”), which corresponded to the an RTC product, was also under a 30% CO_2_ and 70% N_2_ atmosphere, with the addition of CH at 1.5% (vol/wt) and T essential oil at 0.2% (vol/wt). Consequently, in the present study, “M” and “M-CH-T” denote RTC chicken samples, both stored under a modified atmosphere, in the absence (“M”) and presence of CH/T (“M-CH-T”), respectively.

### 2.6. Enumeration of Pathogenic Bacteria in the RTC Product during Storage

At predetermined time intervals (days 0, 1, 2, 3, 4, 5, 6, 7, and 8), 25 g of RTC product from each treatment was added to 225 mL of 0.1% (wt/vol) buffered peptone water (pH = 7.0) (Merck, Darmstadt, Germany) in sterile Stomacher bags. Samples were homogenized for 60 s in a Stomacher (Seward Medical, Worthing, UK) at room temperature. The suspension was serially diluted in 0.1% buffered peptone water, and aliquots were withdrawn and plated in duplicate. Counts of *L. monocytogenes* were monitored on Agar *Listeria* Ottaviani and Agosti (ALOA) (BioMérieux, Craponne, France) and for *S. Montevideo* on xylose lysine deoxycholate agar (XLD) (Oxoid, Basingstoke, UK), after incubation of the plates at 37 °C for 24 h. The testing method adopted for all EU baseline surveys in poultry was a modification of ISO 6579:2002, consisting of modified semi-solid Rapapport Vassiliadis medium (MSRV) [30] and replacing the use of both Rapapport Vassiliadis Soya and Muller–Kauffmann tetrathionate broth with novobiocin. After pre-enrichment in MSRV, two plating media are suggested, the first one frequently in use is the Xylose Lysine Deoxycholate (XLD) agar and the second medium a choice of the laboratory. Additionally, the mixture of the headspace in each package (O_2_ and CO_2_ concentrations, % vol/vol) was measured using a PBI Dansensor A/S (Check Mate 9900 O_2_/CO_2_; Ringsted, Denmark) analyzer (accuracy: ±0.1%). Approximately 3 mL of gas was sampled from the package’s headspace using a needle. Two samples from each treatment were taken for microbiological analysis on each day of sampling.

### 2.7. Risk Management of the RTC Product

Scenarios based on results from previously published studies [22,23,24,25,26,27], as well as from the predictive models and challenge tests performed in our study, were used in order to define the PO for the RTC product. To confirm compliance with an FSO for the chicken product, the following formula was used [19] to calculate the FSO and PO throughout the shelf-life: Ho − ∑R + ∑I ≤ FSO, where FSO is the Food Safety Objective, Ho is the hazard initial level, ∑R and ∑I represent a total decrease and a total increase (due to recontamination and/or growth) in the hazard on a cumulative basis, ≤ = preferably less than, but at worst equal to, FSO; all values are expressed in log_10_ units. The aforementioned equation expresses the relationship between the “initial level”, “reduction,” “increase”, and the FSO [31]. The experimental data obtained in our study were based on using a microwave as thermal inactivation treatment applied by consumers for RTC products [22,29].

### 2.8. Statistical Analysis

Results are expressed as mean ± standard error. One-way analysis of variance (ANOVA; IBM SPSS Statistics 19.0) showed significant interactions between temperature and microbiological growth of the pathogens. These significant interactions were further interpreted, and Student’s two-tailed t-tests followed by Tukey’s post hoc tests were performed. Statistical significance was set at *p* < 0.05. Three replicate experiments were conducted, and two samples were analyzed for each (*n* = 3 × 2 = 6).

## 3. Results

**Figure 1:**
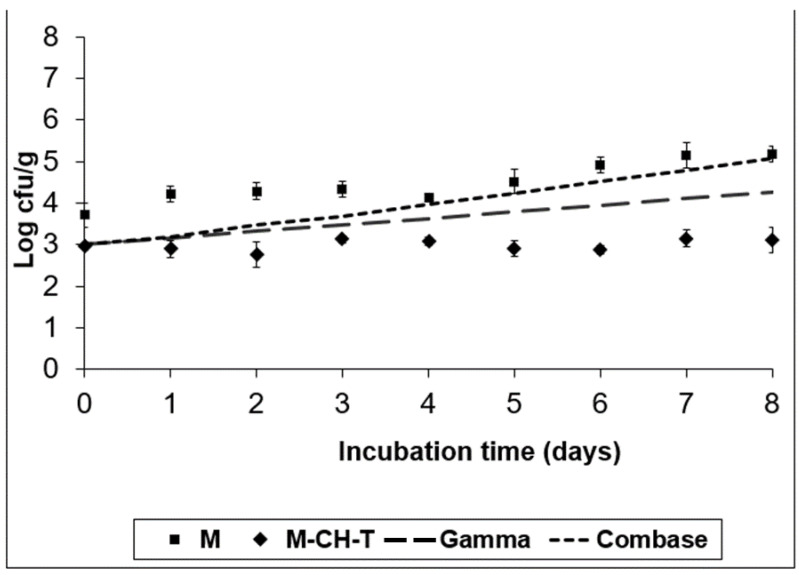
Growth of *L. monocytogenes* on the RTC product stored at 4 °C, under MAP (M; ■), under MAP with Chitosan/Thyme oil (M-CH-T; ♦) using the Gamma prediction model (— —) and the Combase^®^ model (- - -). M denotes RTC product, stored under MAP and M-CH-T a product stored under MAP, treated with Chitosan and Thyme.

**Figure 2:**
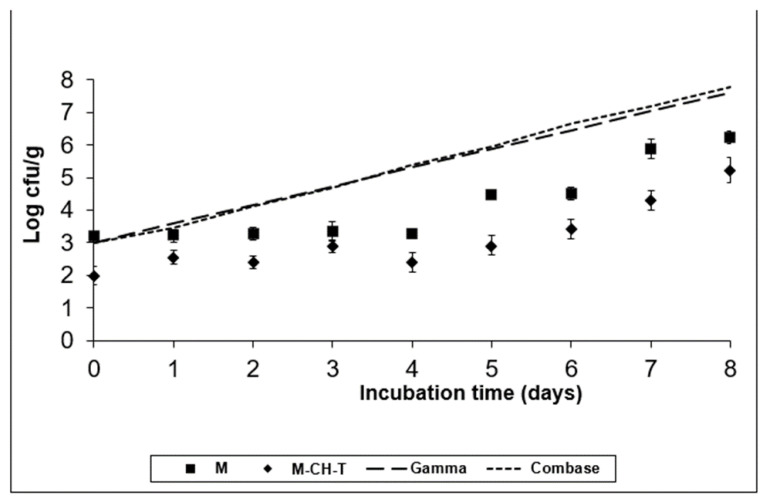
Growth of *L. monocytogenes* on the RTC product stored at 8 °C, under MAP (M; ■), under MAP with Chitosan/Thyme oil (M-CH-T; ♦) using the Gamma prediction model (— —) and the Com-base^®^ model (- - - -). M denotes RTC product, stored under MAP and M-CH-T a product stored under MAP, treated with Chitosan and Thyme.

**Figure 3:**
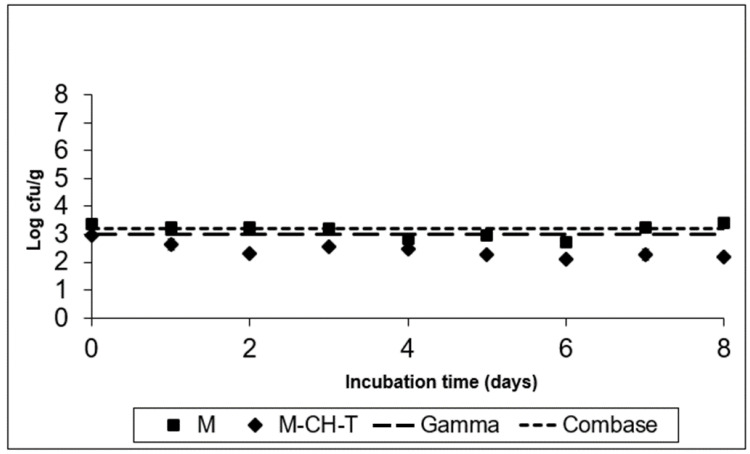
Growth of *S. Montevideo* on the RTC product stored at 4 °C, under MAP (M; ■), under MAP with Chitosan/Thyme oil (M-CH-T; ♦) using the Gamma prediction model (— —) and the Com-base^®^ model (- - - ). M denotes RTC product, stored under MAP and M-CH-T a product stored under MAP, treated with Chitosan and Thyme.

**Figure 4:**
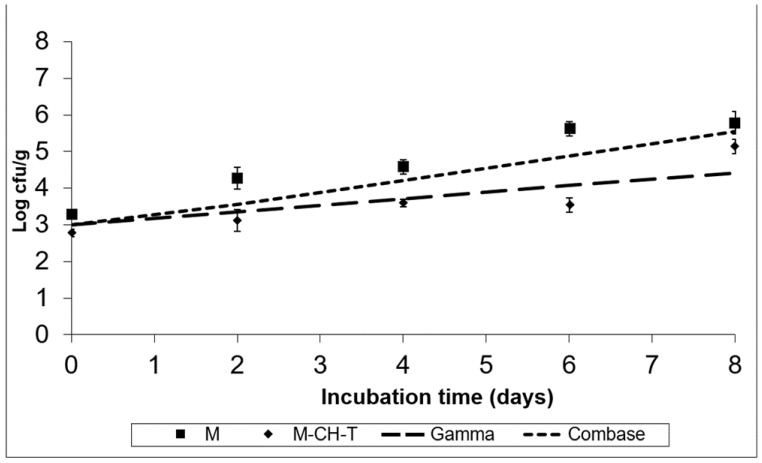
Growth of *S. Montevideo* on the RTC product stored at 4 °C, under MAP (M; ■), under MAP with Chitosan/Thyme oil (M-CH-T; ♦) using the Gamma prediction model (— —) and the Com-base^®^ model (- - -). M denotes RTC product, stored under MAP and M-CH-T a product stored under MAP, treated with Chitosan and Thyme.

A semi-quantitative spreadsheet software was used to facilitate risk management prioritization. The software embodies the established principles of the food safety risk assessment. As a preliminary phase of the Microbiological Risk Assessment for the RTC product, the selection and the importance of the pathogens was based on the ingredients of the product (chicken and bell peppers), its growth characteristics (e.g., pH, water activity, temperature) during storage conditions, the product’s shelf-life, and finally the age of the population that would consume the product. Inadequate hygienic conditions could lead to cross-contamination of the product with pathogens. The results obtained from the risk analyses were used to identify and prioritize foodborne risks related to the consumption of RTC products. Table 1 shows the risk ranking of the pathogens’ selection. The ranking results were evaluated by a group of food microbiologists. Figure 1, Figure 2, Figure 3 and Figure 4 illustrate the growth of the selected pathogens in the RTC product stored under MAP conditions with no antimicrobials (M) added, as well as after being treated with a combination of natural antimicrobials (M-CH-T) at two different temperatures (4 °C and 8 °C). The models used are explained in Section 2.2. The results of the predictive models were validated using the results from the challenge tests. According to Figure 1, Combase^®^ program estimates a growth of ca. 2.0 log CFU/g after eight days of storage at 4 °C of *L. monocytogenes*, while the Gamma model shows a smaller growth (ca. 1.3 log CFU/g) under the same temperature conditions. At the abuse temperature (8 °C, Figure 2), the Combase^®^ model showed a growth of ca. 4.8 log CFU/g of *L. monocytogenes*. Similarly, the Gamma model showed an increasing growth of the pathogen at temperature abuse, which was similar to the one predicted by the Combase^®^ model (difference of 0.16 log CFU/g on d-8 of storage).

According to the experimental data for M treatment at 4 °C (Figure 1), the inoculated pathogen showed an increased growth of approximately 1.5 log CFU/g compared to its initial value (day 0), which was retained until the end of the storage period (day 8). Regarding the data obtained for M-CH-T, it was observed that the combination of chitosan and thyme oil resulted to viable counts of *L. monocytogenes* that remained at stable levels throughout the storage period at 4 °C. The final counts of the pathogen in M-CH-T samples stored at 4 °C were approximately 2.0 log CFU/g lower than the respective counts in control (M) samples. A slow growth of *L. monocytogenes* was noted when M samples were stored at 8 °C, from day 0 until day 6 of storage, showing a 1.3 log CFU/g increase, compared to its initial value (Figure 2). Conversely, after day 6 of storage, *L. monocytogenes* grew faster, reaching levels of approximately 6.2 log CFU/g, showing a 3.0 log CFU/g increase, compared to the initial value of inoculation. Under the same temperature conditions (8 °C), *L. monocytogenes* followed a slower growth pattern for M-CH-T. Values of *L. monocytogenes* counts reached the initial inoculated level of 3.0 log CFU/g after 5–6 days, and on day 8 of storage, the pathogen grew up to 5.2 log CFU/g. *L. monocytogenes* populations on M-CH-T sample were 1.0 log CFU/g lower than the respective counts in the M samples at day 0 (8 °C). This difference of approximately 1–1.5 log CFU/g in the counts of the pathogen populations was noted throughout the storage period at 8 °C. At 4 °C, the Gamma model predictions were closer to the experimental data obtained for the M and M-CH-T samples. During storage at 8 °C, data obtained from the challenge tests on M and M-CH-T samples fell under predictions for both models (fail-safe scenario).

Figure 3 and Figure 4 show the fate of *S. Montevideo* inoculated in treatments M and M-CH-T, stored at 4 °C and 8 °C, respectively. Using the Gamma model, it was predicted that *Salmonella* spp. could not grow at low chilled temperatures (4 °C), although it could survive during the product’s preservation. According to calculations based on the Combase^®^ model, the growth of this microorganism was inhibited at 4 °C. However, at 8 °C (Figure 4), the Gamma model showed a growth of ca. 1.4 log CFU/g at the end of the time of storage, whereas Combase^®^ estimated a higher growth, reaching a ca. 2.5 log CFU/g increase at the end of the storage period. From the comparison of the two models, a difference of 1.1 log CFU/g in the final predicted counts of *Salmonella* was obtained.

The experimental data of *Salmonella Montevideo* survival after inoculation of treatment M, stored at 4 °C presented in Figure 3, showed that the pathogen survived and in fact remained at the initially inoculated counts. However, the application of both CH and T under MAP conditions (M-CH-T) resulted in a final reduction of 1.0–1.2 log CFU/g compared to that in M samples stored at 4 °C. At the abuse temperature (8 °C, Figure 4), the counts increased by 2.5 CFU/g from their initial counts (3.3 log CFU/g), reaching 5.8 logs in the M samples after 8 d of storage. In contrast, the growth rate of *S. Montevideo* in the M-CH-T samples was lower than that in the M samples. From day 0 until day 6 of storage at 8 °C, *Salmonella* counts on M-CH-T reached 3.5 log CFU/g, followed by a sequential increase of 1.6 log CFU/g until final day (8). On day 6 of storage, counts of *S. Montevideo* in the treated samples (M-CH-T) were lower by approximately 2.0 log CFU/g compared to those in the M samples. Additionally, it was observed that extension of the storage time at 8 °C (Figure 4) led to a lower reduction in pathogen growth (0.7 log CFU/g lower than that of M on day 8).

The results obtained from the experimental data at 4 °C for both treatments agree with the predicted results when using the Gamma or Combase^®^ model. In the case of the M samples, the behavior of the microorganisms during storage at abuse temperature (8 °C) agrees better with that of the Combase^®^ model. Treatment M-CH-T showed a slower growth of the pathogen at 8 °C than at 4 °C, and the Gamma model was the one that best predicted the behavior of *Salmonella* spp. at this temperature condition. For *L. monocytogenes* and *Salmonella* spp., the FSOs used were −0.3 for *L. monocytogenes* and −6.7 for *Salmonella* spp., respectively. Data from the challenge tests and the suggested FSOs were used, and Table 2 was constructed based on the methodology described by Mejia et al. [22], showing different scenarios of the PO for both products’ recipes. Initial cell numbers (described on the product) were considered as the initial contamination of the RTC product, and as a fail-safe scenario. In the examples presented in Table 2 for *L. monocytogenes*, we assumed that the microbiological states of raw RTC product (H_o_) is set at 3.0 log CFU/g, and the FSO is ≤−0.3 log CFU/g, while for *Salmonella* spp. the respective H_o_ values were set at 1.0 log CFU/g and the FSO ≤ −6.7, respectively.

## 4. Discussion

Risk Ranger^®^ was a useful tool used to identify the main risks in the product. The Risk Ranking value is scaled logarithmically between 0 and 100, where 0 represents no risk, and 100 represents the highest risk where every member of the population eats a meal that contains a lethal dose of the hazard every day [28].

According to the interpretation of the Risk Ranger, high ranking risks were observed for *Campylobacter/Arcobacter* (58), *L. monocytogenes* and *Salmonella* spp. (55), *Bacillus cereus* (49), and *Clostridium botulinum* (52), while *Staphylococcus aureus* showed moderate risks (35). *Campylobacter/Arcobacter* gave a high-risk score, accounting for these two pathogens, along with *Salmonella* spp., being the leading causative agents of foodborne outbreaks [31]. However, during the production process, distribution, and storage of the RTC product, the suggested temperature is 4 °C; thus, *Campylobacter/Arcobacter* is unable to grow at this low temperature (lower growth limit is about 25 °C [29]. 

*Listeria monocytogenes* and *Salmonella* spp. were able to survive and grow at the refrigeration temperatures used in the challenge tests of our study, justifying the high-risk ranking received after using Risk Ranger^®^. Although *C. botulinum* was identified as a potential risk factor for contaminating the RTC product, its growth ability was not investigated in this study. This was based on the fact that the shelf-life of the product is less than 10 d, which is below the maximal storage time recommended by the Food Safety Standards [32] in order to control its toxin formation. *Bacillus cereus* may represent a possible microbiological hazard for the RTC product as determined by Risk Ranger^®^, but this was not investigated in this study. However, its ability to survive and grow in a product will be an objective of a future investigation.

This study showed that *L. monocytogenes* could grow on the RTC product without antimicrobials at both recommended (4 °C) and abuse temperatures (8 °C). The experimental growth of 3.0 logs observed for the RTC product at 8 °C indicates the fast growth rate of the pathogen, which is rarely expected under real storage conditions of meat or poultry products, and such growth however may be hindered due to the dominant background microflora on the RTC product. A similar observation was also noted by Membre et al. [33], who reported similar experimental growth of *L. monocytogenes* in a pork product during storage of 6–10 days at 8 °C, exceeding the values predicted by the kinetic model applied. The results of the present study show that the addition of CH and T to the RTC product was able to control the growth of the pathogens at 4 °C, given that it was noted that final counts of the pathogen when antimicrobials were applied were 2.0 logs lower, in comparison to that of the control samples on the final 8-d of storage. During storage at 8 °C, although the presence of antimicrobials decreased the final counts of the pathogen by 1.0 log, compared to that of the untreated product, this reduction was lower compared to that in samples stored at 4 °C, indicating that at low temperature conditions (4 °C), application of CH and T had a more pronounced antimicrobial effects against *L. monocytogenes*, compared to the (abuse) temperature set at 8 °C in our study. A similar trend was also observed by Aureli et al. [34], who reported that T reduced the viable counts of *L. monocytogenes* in minced pork meat by approximately 2 log CFU/g at 4 °C, but only by 1 log CFU/g at 8 °C. Fernandez-Saiz et al. [35] studied *L. monocytogenes* growth in a fish soup, and also on a broth model after the addition of 10–80 mg CH films. The results of that study showed that the pathogen was less resistant to biocidal chitosan at 4 °C or 37 °C than at 12 °C.

In a related study, Zivanovic et al. [36] reported that pure CH films reduced the number of *L. monocytogenes* inoculated in bologna slices by 2 logs, whereas CH films with 1% and 2% oregano EO decreased the number of *L. monocytogenes* by 3.6 to 4 logs. Ponce et al. [37] also reported limited inhibitory action of CH film-forming solutions against *L. monocytogenes*, but the antibacterial effect was enhanced after enrichment with rosemary.

The combined application of CH and T reduced the counts of *S. Montevideo* by 1.0–1.2 log CFU/g from the initial levels to the end of the storage period (4 °C). At 8 °C, the addition of antimicrobial compounds resulted in restricted growth of the pathogen during the first 6 d of storage (1.0–2.0 CFU/g lower than that of the control), but after this period a sequential growth occurred, meaning that after day 6th of storage, recovered *Salmonella* cells may develop tolerance toward the antimicrobial effeca of the agents over time. Similarly, Coma et al. [38] observed a decrease in the antibacterial effect of CH films with increasing incubation time, which could be attributed to the bonding of the charged amino groups of CH to the components of the surface of the bacteria, thus making them unable to attach to other cell surfaces. Moreover, according to the study of Hao et al. [39] prolongation of the storage period could lead to a reduction in the antibacterial effect of spice and herb extracts against microorganisms.

Studies so far regarding the application of multi-tools, including Stepwise and Interactive Evaluation of Food Safety by an Expert System (SIEFE), food safety objectives (FSO) hazard analysis critical control point (HACCP), Risk Ranger and predictive microbiology (Gamma model), have led to the conclusion that the most important and critical step during preparation of the meals is the cooking step [21,22,23,24,26,29]. Risk Ranger was applied in order to rank the possibility of the pathogens to grow on the specific RTC food product: *Salmonella* and *Campylobacter* scores were the highest, *Bacillus cereus* the lowest. Risk Ranger was also used to assess the effect of the cooking stage on food safety and confirmed the importance of this process [29]. In another study, two approaches were used: a “top-down” (epidemiological country data) and a “bottom-up” (prevalence and concentration of the pathogen at retail), and upon application of ALOP and FSO models, using *Listeria monocytogenes* in deli meats as a case study, predicted a mean estimated ALOP value of 3.2 cases per million inhabitants per year, whereas the bottom-up approach, gave varying ALOP values and in the range 12–44 cases per million inhabitants per year due to deli meats [26].

Finally, according to Mataragas et al. [24], using the concept of FSO, models, meta-analysis, as well as introducing additional killing steps by holding the final product at an elevated temperature for a certain time period, all of these could lead to better control of the growth of *L. monocytogenes* in fermented sausages [40].

The inhibitory effect of thyme EO against *Salmonella* spp. has been reported in several in vitro studies, and bactericidal activity is dose-dependent [7,9,41]. However, there are also studies that have reported divergent results for the antimicrobial action of CH against *Salmonella* spp. The inhibitory activity of chitosan against pathogens depends on the pH of the food matrix, concentration of chitosan added, or storage temperature [35,42]. Pranoto et al. [43] observed no inhibitory effect of pure CH films on *S. Typhimurium,* but the application of garlic oil (100–400 μL/g) reduced bacterial growth of the pathogen underneath the film. Moreover, Inatsu et al. [44] reported that the addition of CH at 0.1% alone or with hop extract in fermented cabbage reduced viable counts of *Salmonella* enteritidis by 0.7 log CFU/g during storage at 10 °C for 4 d.

The Gamma model best predicted the behavior of *L. monocytogenes* and *S. enterica* during storage at 4 °C. However, *L. monocytogenes* growth in both treatments M and M-CH-T fell under the predictions of the two models at 8 °C. This could be due to the fact that during model development, factors such as interactions between pathogens and members of the spoilage microflora, the structure/composition of the product, etc. are not taken into consideration [45]. On the other hand, the experimental results obtained for *Salmonella* at 8 °C for treatment M did not seem to correlate with the predicted values of the Gamma model, as the actual time required to reach the predicted levels of the pathogens was shorter than the time estimated by the model. In another study, the time required to reach 10^6^ CFU/g counts of *L. monocytogenes* during the storage of chicken nuggets at 10 °C was half the value predicted by the predictive model used [46]. Parameters such as the temperature during storage, the head space gas composition and pH, as well as the background microflora during actual storage of the inoculated samples, may lead to differences between predictive and real-time results [46].

After establishing the boundaries of the safety of the RTC product, it was possible to set the PO to reach the FSO, as also proposed for a steam-chicken meal [22]. Prolonged storage of the commercial product (M), 2 days beyond its shelf-life (8 d) under temperature abuse conditions, would lead to an increase of 3.0 log CFU/g of *L. monocytogenes*. However, treatment of the product with CH and T (M-CH-T) or securing a low initial contamination of raw materials (e.g., 1.0 log CFU/g) will reduce the levels of the pathogen during storage, thus approaching the FSO of −0.3 log CFU/g. The proposed FSO for *Salmonella* in the RTC product is set at a much lower level (−6.7 log CFU/g) than that established for *L. monocytogenes.* This means that even if a low initial post-processing recontamination is estimated for the M-CH-T product (e.g., 1 log CFU/g), the FSO could not be easily achieved, although the process criterion of cooking (microwave) before consumption achieves a −6D reduction. Therefore, with the expected much larger inactivation (>6D), it can be assumed that level is achieved; however, this is difficult to prove. Mejia et al. [22] reported similar results for FSO establishment of *Salmonella* in steam RTE chicken meals, emphasizing the need to apply good quality practices through the production of such meals and, thus, ensure their safety. For industries, to reduce the risk of pathogens growing in foods, interventions should be carefully chosen and added to already existing processes in order to provide additional barriers and therefore enhance the measures taken to eliminate hazards. Factors that need to be taken into consideration are sufficient sanitation practices aimed at reducing cross-contamination, good surveillance of the microbiological status of the food products, improvement of the processes that are essential to control the temperature (cool chain) during distribution and storage, as well as the use of natural antimicrobials during production. Finally, training of the employees and the use of risk communication messages/programs to consumers should also be considered [24,47].

## 5. Conclusions

The Risk Ranger^®^ and the predictive models used (Combase^®^ and Gamma) proved to be valuable tools for the ranking of the pathogens, in relation to the food safety risks during storage of the a Ready to Cook chicken product, and in establishing FSO, which for the specific product. Additional studies on the storage time of RTC chicken products are needed, and data that would help further in refining risk estimates and assisting food manufacturers during distribution. A deeper level of knowledge of the impact of both the initial microbiota numbers and variability of incoming primary materials, and the subsequent degree of food control processing, could affect, and, in certain cases, reduce, the presence of potential food pathogens in high-risk perishable products during refrigerated storage, assuring that a suggested FSO is attained.

## Figures and Tables

**Table 1:** Risk Ranger^® 1^ applied to the RTC product.

Risk Ranger Parameters	*Campylobacter/* *Arcobacter*	*Salmonella* spp.	*Staphylococcus aureus*	*Listeria* *monocytogenes*	Non-Proteolytic *Clostridium botulinum*	*Bacillus cereus*
Hazard severity	Moderate	Mild	Mild	Severe	Severe	Mild
How susceptible is the population of interest?	General	General	General	Slight	General	General
Frequency of consumption	Weekly	weekly	weekly	Weekly	weekly	weekly
Proportion of population consuming product	Some (25%)	Some (25%)	Some (25%)	Some (25%)	Some (25%)	Some (25%)
Size of population consuming product	11,237,068 ^2^	11,237,068 ^2^	11,237,068 ^2^	11,237,068 ^2^	11,237,068 ^2^	11,237,068 ^2^
Probability of contamination of raw product per serving	10% sometimes	10% sometimes	10% sometimes	10% sometimes	10% sometimes	10% sometimes
Effect of processing	No effect	No effect	No effect	No effect	No effect	No effect
Is there potential for recontamination after processing?	Yes-minor 1%	Yes-minor 1%	Yes-minor 1%	Yes-minor 1%	Yes-minor 1%	Yes-minor 1%
How effective is the post-processing control system?	Well controlled	Controlled	Well controlled	Controlled	Well controlled	Controlled
What increase in the post processing contamination level would cause infection or intoxication to the average consumer?	Slight (10-fold increase)	Slight (10-fold increase)	Significant (10,000-fold increase)	Significant (10,000-fold increase)	Significant (10,000-fold increase)	Significant (10,000-fold increase)
Effect of preparation before eating	99%	99%	99%	99%	99% ^3^	No effect ^4^
Probability of illness per day per consumer of interest	1.42 × 10^−3^	4.27 × 10^−5^	1.42 × 10^−8^	2.14 × 10^−7^	1.42 × 10^−8^	4.27 × 10^−6^
Total predicted illness/annum in population of interests	1.46 × 10^4^	4.38 × 10^4^	1.46 × 10^1^	4.38 × 10^1^	1.46 × 10^1^	4.38 × 10^3^
Risk Ranking	58 high	55 high	35 medium	55 high	52 high	49 high

^1^. https://www.fao.org/food-safety/resources/tools/details/zh/c/1191489/ (on 16 June 2022); ^2^. Population in Greece www.statistics.gr (http://www.statistics.gr/portal/page/portal/ESYE (1 March 2021); ^3^. Toxin is heat labile; ^4^. Heating (microwave) will have effect on vegetative cells but not in on the spores.

**Table 2:** Determination of PO and evaluation of FSO in the food chain of the RTC product stored under MAP conditions (commercial product, treatment M), and in presence of Chitosan-Thyme oil (treatment M-CH-T) at recommended chill (4 °C) and abuse (8 °C) temperatures with different scenarios. M denotes RTC product stored MAP, M-CH-T denotes product treated with Chitosan and Thyme, stored under MAP.

Scenario	Pathogen	Treatment	N_o_ Product after Production	∑G Growth at Retail	N_o_/PO Product in the House	∑R	Total	FSO	Accept/Reject Product
1 (4 °C)	*Listeria monocytogenes* (Day 8 ^a^)	M:	3	2	5	>−6	≤−1	−0.3	accept ^b^
		M-CH-T:	3	0	3	>−6	≤−3		accept ^b^
2 (8 °C)	*Listeria monocytogenes* (Day 8 ^a^)	M:	3	3	6	>−6	≤0	−0.3	accept ^b^
		M-CH-T:	3	2	5	>−6	≤−1		accept ^b^
3 (4 °C)	*Salmonella Montevideo* (Day 8 ^a^)	M:	1	0	1	>−6	≤−5	−6.7	reject ^c^
		M-CH-T:	1	−1	0	>−6	≤−6		reject ^c^
4 (8 °C)	*Salmonella Montevideo* (Day 8 ^a^)	M:	1	2.5	3.5	>−6	≤−2.5	−6.7	reject ^c^
		M-CH-T:	1	2.0	3.0	>−6	≤−3.0		reject ^c^

N_o_ = Initial contamination number (Log N/g), G = Growth of pathogen in the RTC product after processing and during storage (Log N/g), N_o_/PO = Performance objective or level of the pathogen before cooking (Log N/g), R = Reduction of the contamination by cooking (microwave) (Log N/g); ^a^ Day 8 = two days after expiration date of the product, ^b^ Considering heating inactivation (microwave) as higher than 6 log CFU/g it was assumed the meals stored at 8 °C for 8 days would be accepted. ^c^ With the expected much larger inactivation (microwave) ≥ 6D, it can be assumed that level is achieved, however is difficult to prove.

## Data Availability

The data presented in this study are available in article.
